# Neural Correlates of Impaired Grasp Function in Children with Unilateral Spastic Cerebral Palsy

**DOI:** 10.3390/brainsci13071102

**Published:** 2023-07-21

**Authors:** Jennifer Gutterman, Andrew M. Gordon

**Affiliations:** Department of Biobehavioral Sciences, Teachers College, Columbia University, New York, NY 10027, USA; jeg2211@tc.columbia.edu

**Keywords:** cerebral palsy, corticospinal tract, sensory, hand, upper extremity, diffusion tensor imaging, dorsal column medial lemniscus, grasp, fingertip forces, sensorimotor integration

## Abstract

Unilateral spastic cerebral palsy (USCP) is caused by damage to the developing brain and affects motor function, mainly lateralized to one side of the body. Children with USCP have difficulties grasping objects, which can affect their ability to perform daily activities. Although cerebral palsy is typically classified according to motor function, sensory abnormalities are often present as well and may contribute to motor impairments, including grasping. In this review, we show that the integrity and connectivity pattern of the corticospinal tract (CST) is related to execution and anticipatory control of grasping. However, as this may not explain all the variance of impairments in grasping function, we also describe the potential roles of sensory and sensorimotor integration deficits that contribute to grasp impairments. We highlight studies measuring fingertip forces during object manipulation tasks, as this approach allows for the dissection of the close association of sensory and motor function and can detect the discriminant use of sensory information during a complex, functional task (i.e., grasping). In addition, we discuss the importance of examining the interactions of the sensory and motor systems together, rather than in isolation. Finally, we suggest future directions for research to understand the underlying mechanisms of grasp impairments.

## 1. Introduction

Cerebral palsy (CP) is a neurological disorder caused by a prenatal or perinatal injury. It is the most common pediatric neurological and physical disability [1,2]. Unilateral spastic cerebral palsy (USCP), one of the most common forms of CP, causes motor impairments that are mostly lateralized to one side of the body. These impairments can affect the ability to perform daily activities [3], particularly grasping (e.g., [4,5,6]).

Most research is focused on the behavioral characteristics of grasping in USCP. However, to understand the mechanisms of the impairment and to improve rehabilitation and upper extremity function, it is important to look at the relationship between brain structure and function. In this review, we aim to summarize our current understanding of the relationship of the development of various tracts in the brain and grasp function. Specifically, we will focus on studies on fingertip force control during precision grip, and the neural correlates once contact with the object is achieved. Precision grip, involving grasping between the thumb and forefinger, is a hallmark of human motor behavior, and is essential to manipulate small objects and skilled tool use. The study of this skill provides a sensitive method to quantify the contribution of the motor and sensory systems to a functional task. The paradigm was first used by Johansson and Westling in a series of studies starting four decades ago to examine cutaneous afferent input during grasp [7,8,9,10]. Since then, this approach has been used in many patient populations, including children with USCP to examine various aspects of grasp such as anticipatory control and adaptation of fingertip forces (e.g., [4,5,6,11,12,13,14,15]).

We will discuss studies on the integrity of the corticospinal tract (CST) and its relationship with grasp impairments in children with USCP. The CST, which is the primary neural pathway for voluntary digit control, is important for the grasping of objects (e.g., [11,16]). Yet other factors such as damage to the sensory tract, CST organization, and damage to the corpus callosum (CC) may also contribute to grasp function. Sensory abnormalities are often present in children with USCP, with approximately 90 percent of children with USCP displaying impaired sensory function [17]. Furthermore, some children with USCP have contralesional reorganization of the CST alongside an intact contralateral sensory tract [18,19,20,21]. This can disrupt sensorimotor integration [20]. The CC is thought to transfer somatosensory information between hemispheres [22,23]. However, children with USCP display structural abnormalities of the CC [24,25]. Thus, when the sensory and motor tracts are in different hemispheres and somatosensory information needs to be transferred between hemispheres, damage to the CC may further contribute to grasp impairments in children with this sensory-motor dissociation. Nonetheless, to the authors best knowledge, the neural underpinnings of the close interaction between sensory and motor tracts on grasp impairments, specifically regarding precision grip, in children with USCP have not been examined. Finally, we will suggest possible directions for future research to fill the gaps in our understanding of the neural basis of grasp impairments in children with USCP.

## 2. Typically Developing Grasp

### 2.1. Neural Mechanisms Underlying Grasp

Animal studies have demonstrated that the control of grasping primarily involves the integration of sensory input with motor output, where the process is mediated by the connection of the prefrontal cortex with parietal areas [26]. In the context of the functional role of the sensory and motor systems, precision grip tasks have been used in numerous studies (e.g., [27,28,29]). Independent finger movement is controlled by the motor cortex via the CST, which is important for object manipulation (e.g., [28,29,30,31]). Lesions in the motor cortex show ablation of independent finger movements [29] and sensorimotor deficits [28]. A greater number of corticospinal neurons were activated during a precision grip task, in which independent finger movements of animals were used, compared to a power grip in which independent finger movements were not used [32]. Similarly, somatosensory information is transmitted through the dorsal column medial lemniscus (DCML) pathway and is also important for grasping and discrimination of tactile information during manipulation of objects [33]. Lesions of sensory pathways result in motor deficits, such as lack of coordination of the index finger and thumb and instead, the whole hand is used to manipulate an object [27]. Further, neuronal activity was increased in the somatosensory cortex of monkeys around grip onset during a precision grip task [34], and there was activity of neurons in the somatosensory cortex when there was a change of friction between the fingertips and object surface [35].

### 2.2. Grasping in Typically Developed Adults

Precision grip lifts in human studies also demonstrate the importance of the motor cortex, as it is shown that the strongest amplitudes of electromyography (EMG) responses are right before the grasp of an object, and on the onset of the manipulation of the object [36]. Damage to the corticospinal connections from the motor cortex to the hand muscles causes impairments in precision grip [37,38].

**Figure 1:**
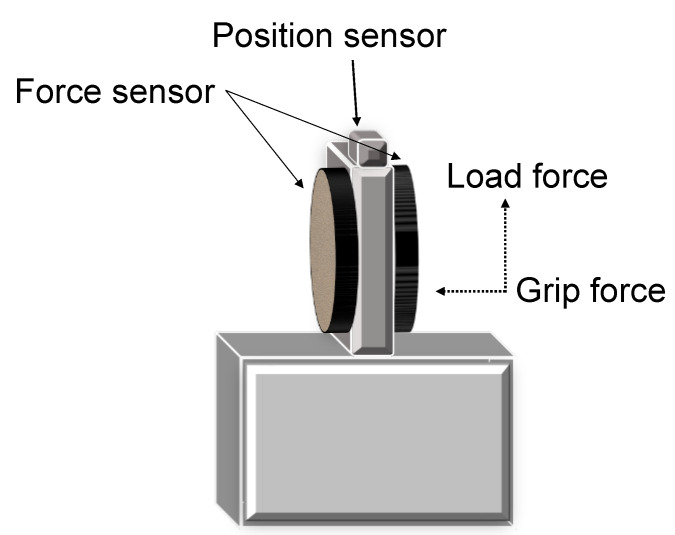
A custom-made device with force transducers and a position sensor used for the lift-grip task. Interchangeable surfaces on the left and right sides of the force sensors. Shaded box represents the area with an exchangeable mass.

**Figure 2:**
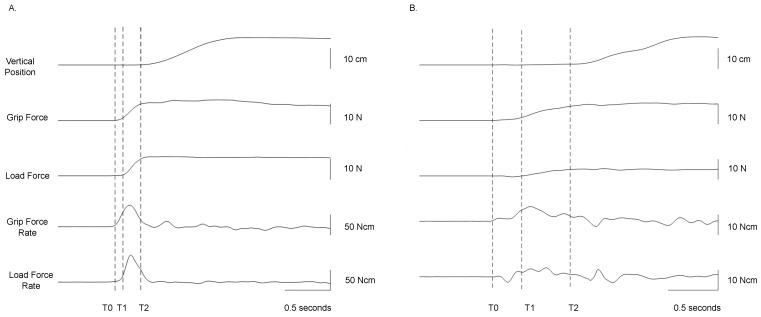
(**A**) A representative trace for a typically developing adult grasping an object that is 910 g; In T0–T1, there is an increase of GF (preloading phase) prior to onset of the load (lifting) force. T1–T2 represents the loading phase where there is a parallel increase of GF and LF, with unimodal peak rates of GF and LF until liftoff. (**B**) A representative trace for a child with unilateral spastic cerebral palsy grasping an object that is 470 g. In T0–T1, there is an increase of GF with negative LF and small peak force rates. T1–T2 peak force rates with multiple peaks until the object gets lifted. GF = grip force; LF = load force.

The study of fingertip forces during precision grip has been instrumental to our understanding of sensorimotor control of prehension. These studies require subjects to lift an instrumented object. Figure 1 shows a typical device used to examine precision grip. There are force transducers below the grasp surface that measure the grip (normal) force (GF) and the load (vertical lifting) force (LF) at the fingertips. A position sensor tracks the object’s location. Further, there are interchangeable surfaces (i.e., sandpaper or rayon surfaces) allowing the study of tactile adaptation to the object’s texture and an area with an exchangeable mass allowing the study of fingertip force adaptation to object mass. To successfully lift an object, subjects must develop sufficient GF and then LF until the LF overcomes the gravitational force on the object and it is lifted off its surface. A representative trace of the grip lift task in a typically developing adult is shown in Figure 2A. The task is denoted by several temporal phases. The preload phase (T0–T1) is the duration between contact with the object and the onset of positive LF. In this phase, there is an initial GF increase to establish a secure grip on the object. After this, during the loading phase (T1–T2), which occurs from positive LF until lift-off (T2) of the object, there is a synchronous increase of GF and LF (i.e., grip-lift synergy), with single unimodal peaks of GF and LF rates. After lift-off of the object, feedback of the object’s mass is available, and the object is held stable in the air in the static phase. The feedback during the static phase is used to adapt the fingertip forces to the object’s texture and weight. The GFs must be sufficiently large enough as to prevent slips, yet not so large as to cause fatigue or crushing fragile objects. This depends on precise integration of tactile and proprioceptive information. Reception of somatosensory information and having the ability to recognize different stimuli by touch is shown to be necessary for the manipulation of objects [39]. This can particularly be seen during this task, where digital anesthesia leads to prolonged temporal phases and a loss of adaptation of forces to object texture [8,10] and higher GFs [8,40,41].

Successful manipulation requires the interplay between anticipatory (motor planning) and feedback control. Visual information is used to identify previously manipulated objects or estimate the required fingertip force scaling based on size, shape, and density cues to drive the manipulation of the object [42,43,44,45]. Higher forces are used for more slippery or heavier objects [7,8,9]. It is important to scale the forces in advance according to the object properties for a more efficient grasp control [7,8,9,42]. Such anticipatory control, based on internal representations is essential given the delays before feedback becomes available. The internal representation is updated based on sensory feedback after each lift, to be used for subsequent manipulation with that object [7,8,9].

## 3. Development of the CST and Grasp in Typically Developing Children

During typical development, the CST approaches the spinal cord by the 20th week of gestation [46] and continues to myelinate until the first years of life [47]. The CST connections have bilateral crossed connections [48]. Over time, the majority of ipsilateral (uncrossed) connections get pruned away, leaving mostly contralateral projections from the motor cortex [48]. This results in a predominant contralateral control of voluntary motor movements. Roughly, around the same time that the CST develops, precision grip emerges, occurring at approximately 10 months in typically developing (TD) children [31,47,49,50]. Before the age of two, TD children display an uncoordinated grasp control, including long temporal phases, high GF at LF onset, asynchronous force development that does not reflect prior experience with the object, large variability with high GFs compared to older children and adults (see Gordon, 1994) [50]. This demonstrates that young children rely more on a feedback strategy rather than a feedforward (anticipatory) control strategy [51]. Children around two years of age start to use sensory information from prior manipulatory experience with an object to scale objects in an anticipatory fashion based on its weight [52] and texture [53]. Precision grip skills continue to develop over time until they approximate adult performance at six to eight years of age [51,52].

## 4. Grasp Impairments in Children with USCP

CP is the most common physical and neurological childhood disability, affecting approximately 2 per 1000 births [1,2]. The most common form of CP, USCP (commonly referred to as hemiplegic CP), primarily affects one side of the body, including upper limb function [1]. This leads to activity limitations [3], difficulty manipulating objects (e.g., [5,6,13]), sensory impairments (e.g., [54,55,56]), weak grip strength (e.g., [16,57,58,59]), and spasticity (e.g., [6,60]). Manual dexterity impairments, tested using timed object manipulation tasks, such as the Jebsen Taylor Test of Hand Function (JTTHF) [61] and the Box and Blocks (B&B) test have been identified in this population [62]. Upper limb sensory impairments have been demonstrated with psychophysical discrimination tasks such as the child adapted grating orientation task (GOT) [63] adapted from Van Boven & Johnson (1994) [64], stereognosis [65] and two-point discrimination tests [66,67]. Sensory abilities have been shown to be important for manipulation of objects [5,6,68].

The relationship between these motor and sensory upper extremity impairments has been explored as well. For example, better sensory function related to greater unimanual capacity, as measured through the Melbourne Assessment (MA), which includes a grasp component [54]. Likewise, the poorer the sensory function, the greater impairments in manual dexterity in children with USCP [69]. However, no relationship was demonstrated between discrimination threshold, as measured by the GOT and dexterity [70]. Interestingly, children with worse baseline manual dexterity prior to training showed improvement on the stereognosis test score after training [71,72], but not with the GOT [72]. Together, these studies suggest a relationship between sensory and motor function regarding stereognosis scores, albeit with mixed results with other measures.

### 4.1. Precision Grip Impairments in Children with USCP

Precision grip impairments are present in children with USCP (e.g., [5,6,12,13,60]). A representative trace of a child with USCP is shown in Figure 2B. Children with USCP exhibit a prolonged preloading phase, a high GF at LF onset (T1), slow, sequential, and asynchronous development of fingertip forces during the loading phase, with multiple force rate peaks (T1–T2). After lift-off (T2), children with USCP display reduced adaptation to object properties [12,13].

#### 4.1.1. Sensory Adaptation Impairments

Sensory impairments may affect grasp function in children with USCP, [5,6,13,60] as impaired sensory feedback may result in an inability to correct online movement errors to modify their force output according to the object properties (i.e., texture and weight). However, there are some discrepancies regarding adaptation to object texture and weight during a precision grip task. Children with CP display excessive GF [13], which is similar to adults grasping objects with digital anesthesia [8,40,41]. Children with bilateral and unilateral CP display a higher safety margin compared to TD children and require repeated trials to adapt the GF to the object texture in the static phase [13]. In contrast, it has been found that children with USCP do not have a higher safety margin or higher static GF than TD children [5], although the extent of adaptation is extremely variable in children with USCP [6].

Children with unilateral and bilateral CP have variable static GF, with large safety margins in comparison to TD children [4]. When lifting objects of different weights, it has been shown that although most children with unilateral and bilateral CP modulate the GF according to the object’s weight during the static phase, static GF was excessive and more variable than the force produced in TD children [12]. Additionally, the modulation of GF to object weight was less prominent than with TD children. Further, it has been demonstrated that children with USCP produced higher forces for heavier objects compared to lighter objects, with a similar amount of GF in the static phase as TD children [5]. However, children with USCP had greater variability in their forces compared to TD children [5]. The discrepancies in some of these studies may be due to participants’ ages and the subtype of CP, as well as the heterogeneity of the small samples. Eliasson et al. (1991) and (1992) included children with unilateral and bilateral CP, six to eight years old [4,12]. This age group in children with CP displays similar grasp impairments to TD young children just learning to grasp [12]. However, Gordon and Duff (1999a) included a wider age group of children and only children with USCP [5].

Another aspect of manipulation of objects is the release phase of objects. Children with USCP demonstrate prolonged and uncoordinated release of objects [60], which may be due to insufficient tactile input to release the object in a fluid motion, as afferent information signaling object contact with the table surface has shown to be important when releasing fingers slowly from an object [7].

Relationships between sensory impairments on clinical tests were measured in relationship to the precision grip task in children with USCP. Gordon and Duff (1999b) found a relationship between poorer sensory function, as measured through stereognosis and less adaptation of static GF during the precision grip task [6]. Further, poorer tactile sensibility, as measured through the two-point discrimination test, related to poorer grasp task performance as measured by less adaptation of static GF [6] and a longer time duration of the release of each finger from an object [60]. However, Eliasson et al. (1995) did not find a relationship between sensory function and adaptation of static GF [13]. The discrepancy may be due to that Eliasson et al. (1995) included children with bilateral CP in addition to children with unilateral CP [13]. Overall, these studies indicate that tactile sensibility may be important for grasp performance. However, other features of CP, such as spasticity, may contribute to the execution of grasping including less adaptation of fingertip forces [6] as well as a prolonged release phase of an object [60].

#### 4.1.2. Anticipatory Control Impairments

Children with USCP display anticipatory control impairments during grasp (e.g., [5,12,15,73]. There are ‘global planning’ impairments in anticipatory control of action during functional tasks where anticipatory control impairments are displayed in both the more and less affected hands (e.g., [15,73,74]). For example, the manner and location of which they grasp the object with their less affected hand may not be biomechanically optimal for keeping an asymmetrical object from tilting after it is lifted, as they keep their thumb collinear or lower than their index finger [15]. However, the scaling of force development is effector dependent, as the impairments are lateralized to the more affected hand ([11,68,75,76]). Specifically, children with USCP do not scale their forces in advance to object weight [5,12] or texture [5,13] with the more affected hand. When children with USCP are provided with extensive practice though, anticipatory control impairments can be partially improved [5,77]. However, they do use a greater proportion of their maximum force compared to TD children since children with USCP have less strength than TD children [60,75]. Further, children with USCP show better anticipatory control of object weight for familiar objects than for novel objects [77]. In addition, children with USCP demonstrate asynchronous forces (i.e., grip-lift synergy) during lifts with their more affected hand as well as a dissociation between gait-induced LF oscillations and GF oscillations while walking with a hand-held object ([76]). Similarly, children with bilateral CP show an impaired grip-lift synergy while lifting an object [78]. Further, some of these anticipatory control impairments may be impeded by spasticity [6]. For example, the more spasticity a child displays, the longer the pre-load duration, reflecting delays between onset of grip and load forces [6]. Although motor skills are shown to emerge with development in TD children [51], children with USCP display similar anticipatory control impairments seen in TD children one to two years of age [4]. This implies that anticipatory control impairments in children with USCP are due to the early lesions that occur.

#### 4.1.3. Sensorimotor Integration Impairments

Sensorimotor integration may play a significant role in grasping impairments in children with USCP [14]. Although children with USCP display anticipatory control (motor planning) impairments with successive lifts with their more affected hand [14,68], anticipatory control emerges on the first lift with their more affected hand after grasping an object with their less affected hand [68]. This indicates that children with USCP may not be getting sufficient sensory information from their more affected hand to sufficiently update the internal model of the object unless they receive enough practice with the object. Interestingly, despite the impaired sensory function and anticipatory control in the affected hand, after lifts with the more affected hand, anticipatory control is immediately seen in the less affected hand [14]. This implies that despite impaired sensory input, the input is sufficient to update internal models of the object. Thus, anticipatory control impairments in grasping are unlikely solely due to impaired sensory input and are more likely due to sensorimotor integration impairments (i.e., integration of sensory and motor information from the same hand). Nonetheless, it should be acknowledged that other factors such as the impaired force control and spasticity may also contribute. The latter may affect the fidelity of proprioceptive input from muscle spindles.

## 5. Neural Basis of Grasp Impairments in Children with USCP

### 5.1. CST Development in Children with USCP

Children with USCP do not always have the same CST development as TD children, as the type and timing of the lesion can cause the CST to reorganize. There are two main types of lesions found in children with USCP. One is periventricular (PV) lesions, which occur in the early third trimester of pregnancy and affect the white matter [79,80]. The other, middle cerebral artery (MCA) lesions, occur in the late third trimester in the brain, are usually larger, and affect the cortex [79,80]. The lesion is shown to result in damage to 133 areas of the brain in children with an MCA lesion and 107 areas of the brain in children with a PV lesion [81]. In particular, several of these areas involve motor control [81]. Some of these areas, such as the cerebellum, basil ganglia, and posterior limb of the internal capsule, are also involved in precision grip control, as this task has been shown to be affected in patient groups with damage to these areas (i.e., Parkinson’s disease, Huntington’s disease, and stroke) [38,82,83,84].

**Figure 3:**
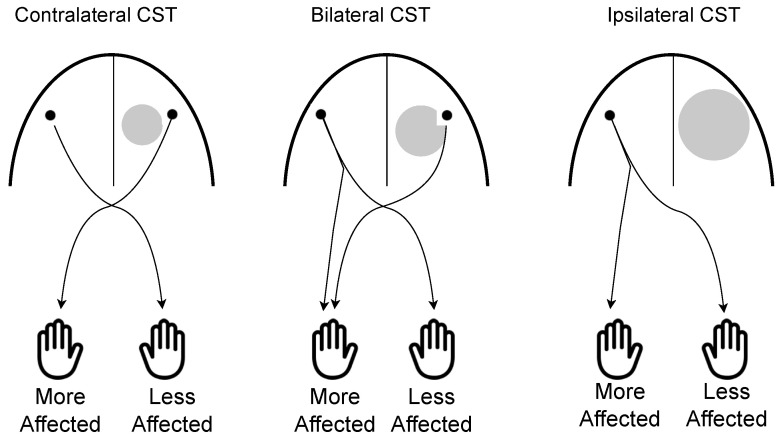
Schematic overview of the corticospinal tract connectivity patterns in children with unilateral spastic cerebral palsy. In the contralateral corticospinal tract (**left**) subgroup, the more affected hand is controlled via crossed projections. In the bilateral corticospinal tract (**middle**) subgroup, the more affected hand is controlled via both crossed and uncrossed ipsilateral projections. In children with an ipsilateral corticospinal tract (**right**), the more affected hand is controlled by the uncrossed ipsilateral projection from the contralesional hemisphere and there is an absence of connections from the lesioned hemisphere.

Though lesions in the developing brains of children with USCP can cause a disruption of development, the CST is capable of considerable reorganization after damage [85]. There are three different CST connectivity patterns in children with USCP that can occur (see Figure 3) [79]. One is the contralateral CST found in TD children, with mostly crossed connections from the lesioned hemisphere [48]. They can also have ipsilateral projections, where the input comes from the uncrossed, dominant hemisphere [46]. Finally, they can have bilateral CST projections, where the connections are from both the dominant and more affected hemispheres [79,80]. It has been shown that use-dependent competition is extremely important in determining organization of the CST [31,86]. The gradual weakening of ipsilateral projections and the strengthening of contralateral projections via synaptic competition during development [46] are driven by motor cortex activity [86].

### 5.2. Damage to the CST in Relation to Grasp Impairments in Children with USCP

Damage to the CST in relation to grasp impairments have been measured in a variety of ways. For example, structural damage has been measured by examining the ratio of the size of the asymmetry of the cerebral peduncles (the more affected peduncle area divided by the less affected peduncle area × 100), in which the CST passes through [11,16,87,88]. The more structural damage to the CST connectivity to the more affected hand muscles, the smaller the ratio (and, thus, the greater the asymmetry) of the CST. Manual dexterity is shown to be associated to the asymmetry index of the CST [87,88]. The greater the asymmetry of the CST, the worse the manual dexterity scores are in children with USCP [87,88].

Diffusion properties of the CST are also related to manual dexterity [89,90], and unimanual capacity [90,91]. Reduced structural integrity (more damage) to the CST, as measured with fractional anisotropy (FA) and radial diffusivity (RD) symmetry index (FA/RD ratio of the more affected to the less affected hemisphere), is associated with poorer unimanual capacity [91]. Similarly, it has been found that reduced structural integrity of the CST in the more affected hemisphere is related to poorer unimanual capacity of the more affected hand in children with USCP [90]. In addition to poorer unimanual capacity, worse dexterity of the more affected hand is shown to be related to reduced structural integrity of the CST in the more affected hemisphere [90]. Nevertheless, a relationship was reported between reduced structural integrity of the CST of the more affected hemisphere and worse manual dexterity of the less affected hand, but not manual dexterity of the more affected hand [89]. In agreement, another study exhibited a relationship between worse dexterity of the less affected hand and reduced structural integrity of the CST in the more affected hemisphere [90].

In addition, transcranial magnetic stimulation has been used to examine the overlap of the primary motor cortex motor representations of both hands [92]. Moderate associations were found between size and excitability of the overlap of the motor representations of both hands and manual dexterity of the more affected hand, with the greater the overlap of the motor representations, the better dexterity of the more affected hand [92].

Grasp impairments relate closely to CST damage, as investigated through performance on a precision grip lift task [11,16]. For instance, the asymmetry of a cross-sectional area of the cerebral peduncles and performance of the grip lift task were examined [16]. An association between the asymmetry of the cerebral peduncles and precision grip performance was found. Children with USCP with more asymmetry exhibited higher GF at LF onset, asynchronous rates of LF and GF and a longer preloading phase (i.e., the duration of the first contact of a digit on the object until the onset of a positive LF) [16]. A similar finding was observed in an LF perturbation task, where the greater the asymmetry of the cerebral peduncles, the greater the temporal impairments of anticipatory control [11]. This was demonstrated by a larger variation in the duration between the LF perturbation and GF max, indicating anticipatory control impairments. Overall, these studies indicate the importance of the CST for precision grip tasks, especially for anticipatory control. However, the CST may not account for all the variance in precision grip impairments in children with USCP, for example the extent of lesions correlated with grip-lift synergy, suggesting grasp function involves several areas in the brain [93]. Therefore, it is crucial to examine other factors, including but not limited to the relationship between the CST and sensory tract location.

### 5.3. Lesion Type and CST Organization in Relationship to Grasp Impairments

Children with PV lesions have better manual dexterity than those with MCA lesions [81,94]. Similarly, it has been demonstrated that the earlier the onset of the lesion, the better hand motor function, measured through a finger opposition test [80]. Children with USCP with a PV lesion display less of a relationship to grasp function compared to children with MCA lesions [57]. The association, therefore, may be more evident in those that have worse grasp function, and other factors, such as CST organization, may influence the differentiation of the relationship to grasp function in children with PV lesions.

CST connectivity patterns, determined by transcranial magnetic stimulation and/or functional magnetic resonance imaging, have shown to contribute to grasp function [59,69,95]. Although there is variability, children with ipsilateral connectivity have greater hand impairments. Factors such as the timing of the lesion [80] and possibly the timing and intensity of rehabilitation may affect motor function [92,96]. Further, it has been established that children with USCP with large PV lesions have ipsilateral CSTs while those with small PV lesions have contralateral CSTs [95]. Children with contralateral CSTs have less severe hand motor impairments on average, shown through a finger opposition task, compared to those with larger lesions who have ipsilateral CSTs [95]. CST wiring also contributes to grip strength and manual dexterity [59]. In agreement, another study found that children with contralateral CST organization display better scores on manual dexterity tests than children with ipsilateral or bilateral CST organization [69].

### 5.4. Development of Sensory Tract in Children with USCP

**Figure 4:**
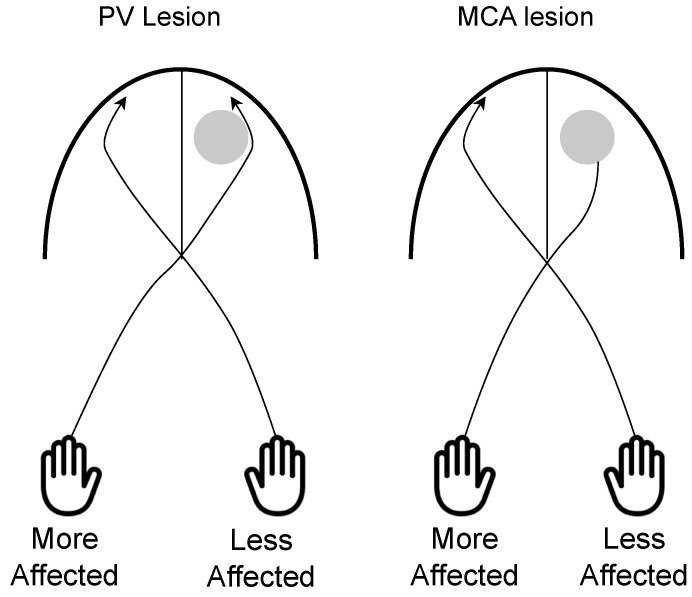
On the (**left**) is a schematic of a child with a periventricular (PV) lesion and on the (**right**) is a schematic of a child with a middle cerebral artery (MCA) lesion. The dorsal column medial lemniscus pathway for each lesion is shown in the representation. PV lesions occur earlier, and, therefore, axons can navigate around the lesion to reach the somatosensory cortex. MCA lesions occur later and are less likely to be able to navigate around the lesion to reach the somatosensory cortex.

In children with USCP, the somatosensory system has a limited potential for reorganization after injury compared to the CST, as reorganization occurs in the ipsilesional hemisphere and does not reorganize to the contralesional hemisphere [18,97]. In those with PV lesions, thalamocortical projections are often still incomplete and, therefore, axons can navigate around the lesion to reach the somatosensory cortex [19]. However, the thalamocortical projections reach their cortical termination sites by the third trimester and, therefore, those with MCA lesions, which occur later than PV lesions, affect the somatosensory system [46] (See Figure 4). Consequently, those that have PV lesions demonstrate better sensory function than those with MCA lesions [21,59].

### 5.5. Sensory Tract Integrity in Relation to Grasp Impairments in Children with USCP

Structural connectivity of the DCML pathway and thalamocortical pathways are shown to relate to grasp function [58,94,98,99]. Those with intact DCML pathways demonstrate better manual dexterity [94]. Reduced structural integrity of the DCML, as measured through mean diffusivity (MD) of the lesioned hemisphere of the DCML and the asymmetry index of the MD, relates to a smaller GF ratio between hands measured with a dynamometer, less muscle strength, and increased muscle tone [58]. Further, increased connectivity of the thalamocortical projections to the somatosensory cortex relate to better manual dexterity [99]. In contrast, another study examining the asymmetry index of the number of fibers within the thalamocortical projections to the somatosensory cortex does not demonstrate correlations with manual dexterity [98]. However, the asymmetry index of the number of fibers within the thalamocortical projection to the primary motor cortex demonstrates correlations with manual dexterity, with the smaller ratio (more damage) relating to worse dexterity [98].

### 5.6. Relationship of Sensory and Motor Tracts on Grasp Impairments

Although many studies have examined sensory and motor tracts in isolation, others examined them simultaneously. However, it is important to examine these tracts together, to gain a better understanding of the contributions of each. Overall, there is conflicting evidence on which tract relates more to grasp function. For example, the presence of somatosensory tracts have a stronger relationship with grasp function in comparison to the absence or presence of the CST, as shown by the larger effect sizes [94]. This indicates increased strength of the relationship between the presence of somatosensory tracts and better grasp function compared to the CST. Further, the larger the asymmetry index of the number of fibers in the sensory pathways to the primary motor cortex (less damage), the better the grasp function, as compared to the CST [98]. Although these studies point to sensory function as the driving factor for function in children with USCP, the presence/absence of the tracts [94] and the number of fibers was examined to look at the relationships [98]. Neither of these studies examined the microstructure of the pathways. However, one study did report the microstructure of the CST, DCML, and superior thalamic radiations [58]. The motor and sensory pathways show moderate correlations with muscle tone, strength, and manual dexterity, with more damage to the pathways relating to increased muscle tone and weakness and worse manual dexterity. These results led to the conclusion that the sensory and motor tracts are both important for grasp function [58].

### 5.7. Relationship between Sensory and Motor Information and the Corpus Callosum in Relationship to Grasp in Children with USCP

Children with USCP may have an inability to appropriately integrate somatosensory information with the motor output of the same hand. Therefore, while they may be able to form an internal representation of the object, the ability to establish the sensorimotor associations used with the motor output in the more affected hand may be impaired. This in part may be due to that for children with ipsilateral CST connections, their sensory information is from the maintained contralateral sensory tract, but they have ipsilateral CST connections (interhemispheric sensory-motor dissociation) [18,20]. In this case, the information would need to be transferred through the CC. The CC is shown to transfer somatosensory information between hemispheres and it is indicated that in patients with an absence of a CC (either congenitally or through surgery) that the CC is vital in transferring somatosensory information from one hemisphere to another [22,23]. Accordingly, the CC may transfer the sensory information between the hemispheres in children with USCP when they have the sensory tract in one hemisphere, but the motor tract in another.

The interhemispheric sensory-motor dissociation and the CC are shown to relate to grasp function. The dissociation of sensory and motor tracts can impact motor impairments [18,19,20,21] and potentially grasp impairments as the dissociation can disrupt sensorimotor integration [20]. Additionally, if there is damage to the CC this could further restrict sensorimotor processing in children with a sensory-motor dissociation, which can have negative effects on upper limb hand function, including manual dexterity of the less affected hand [25]. Greater damage to the CC can also cause functional changes in the unaffected hemisphere [100]. Greater damage to the motor area of the CC is associated with worse manual dexterity in the more and less affected hands [101]. Further, worse manual dexterity moderately relates to the reduced structural integrity of the CC [24]. Therefore, although the motor and sensory tracts show relationships with grasp impairments in children with USCP, the impairments may not be a sensory tract or motor tract problem. Rather, it may be due to how the somatosensory input is integrated with the motor output, which in some cases may involve the CC.

## 6. Limitations and Future Directions

One limitation of previous research in understanding underlying mechanisms of grasp impairments is that the studies involving the integrity of sensory tracts solely used clinical measures. There may be a dissociation between sensory perception and utilization. Furthermore, these tests require the ability to understand the instructions and sufficient attention to detect subtle discrimination levels, and, thus, may not be appropriate for young children or children with cognitive or attention deficits. No studies to date have examined the relationship between integrity of sensory tracts and modulation of fingertip forces during grasping, which is shown to be a reliable, objective, and sensitive measure to examine utilization of sensory information during grasp. This is important as although it is speculated impairments in adaptation of fingertip forces is due to sensory feedback [5,6,13,60], motor impairments may also contribute. Examining the relationship between sensory tracts and this specific task may give us more insight on the mechanisms of adaptation of fingertip forces impairments. Further, many of the studies examining DTI measures used different methodologies and outcome variables, and, therefore, the studies are not directly comparable. More replicability in future studies should be performed to get a better understanding of the relationship between structural integrity of sensory and motor pathways and grasp function. In addition, many of the studies only examined one tract in relation to grasp function. In the future, additional research is needed to examine the sensory and motor tracts together in relation to grasp function to fully understand the contribution of each tract in relation to grasp impairments in children with USCP. Future research should also examine other factors that may contribute, such as lesion size, type of lesion, spasticity, and CST organization, together with these tracts to help understand the contribution of each on the variance of grasp performance in children with USCP. Although all or many of these factors may contribute, it is unlikely one single variable causes impairments in grasp function. For example, if anticipatory impairments of grasp function were purely a motor execution problem due to not being able to control the amount of force and due to spasticity, grasping an object with the less affected hand first, and then the more affected hand, would not remedy the impairment. Nonetheless, with enough practice, or with the use of the achieved sensory inputs with the less affected hand, and thus with the formation of more distinct internal object representations, children with USCP can scale forces appropriately [14]. This suggests that impaired force output and spasticity are not the primary source of impaired anticipatory force control. Furthermore, the examination of CST organization in relation to precision grip studies should be performed. The CST of the lesioned hemisphere in children with ipsilateral organization and the DCML of the less affected hemisphere in children with ipsilateral and bilateral organization is shown to have more structural damage in children with contralateral CST organizations [58]. Therefore, CST organization may additionally contribute to structural integrity of the pathways and, thus, to grasping as well. However, as CST organization was a stronger predictor of the variance of bimanual than unimanual performance, it is postulated that CST organization may play a stronger role in bimanual grasping than unimanual [58]. Future studies should examine differential contributions of the tract integrity and CST organization on bimanual grasping.

There may also be a differentiation in motor planning versus execution in relation to different brain areas. While the CST is shown to be associated with anticipatory motor control impairments [11,16], sensory tracts may be more related to feedback aspects of grasping and the ability to update the internal model. As it is established that the DCML is important for novel objects [102], it may take more trials for children with USCP to form the internal representation needed to grasp the object appropriately in an anticipatory fashion according to the object properties [77]. While one study did examine the symmetry of CST with various aspects of the grip lift task [11], future work should examine the contribution of both the sensory and motor tracts with the various aspects of the grip lift task (i.e., motor planning versus motor execution).

Finally, it has been demonstrated that children with USCP can transfer sensory information signaling object weight from the more affected hand to the less affected hand for use in anticipatory control [68]. Therefore, sensorimotor integration could play a role in grasping impairments in children with USCP. However, previous research with transfer of anticipatory control of forces in children with USCP have only been done with weight, and not texture. Consequently, the transfer of sensory information according to various textures should be examined in future studies with the potential differential effects of the transfer of texture and weight information in children with USCP.

## 7. Conclusions

We described sensory, motor, and sensorimotor integration impairments in children with USCP in relation to grasping, with an emphasis on precision grip studies. We also described developmental differences in the sensory and motor systems. It was demonstrated that the integrity of the CST tract and symmetry of the CST are related to grasp impairments in children with USCP. It was also shown that while there are no studies to date examining the sensory tract and grasp impairments using the precision grip task, children with USCP share many of the same grasp characteristics of adults with experimental suppression of afferent information. Further, we examined the interactions on grasp function between the motor and sensory systems when the tracts are in different hemispheres. The interactions between the motor and sensory systems are complex and it is likely they both play an integrative role in grasp impairments in children with USCP. In addition, it was determined that the CC is important for the transfer of sensory information and that children with USCP can transfer anticipatory control of forces of object weight from the more affected hand to the less affected hand. This implies that anticipatory control impairments in children with USCP are due to sensorimotor integration problems. Overall, it is shown that the development of motor tracts, sensory tracts, and CC play a role in grasp function in children with USCP. Therefore, although children behaviorally display what looks like somatosensory impairments causing insufficient internal models and motor output impairments, the inability to integrate the somatosensory input with the motor output may be what is contributing to anticipatory planning impairments of grasp in children with USCP.

## Data Availability

Not applicable.
